# 

**Published:** 1838-10

**Authors:** 


### BOOKS RECEIVED FOR REVIEW.

### ENGLISH.

1. The Village Pastor's Surgical and Medical Guide: in Letters from an old Physician to a young Clergyman, his Son. By Fenwick Skrimshire, M.D., Physician to the Peterborough Infirmary.—London, 1838. 8vo. pp. 425. 8s.

2. A Manual of the Diseases of the Eye; or, a Treatise on Ophthalmology. By S. Littell, M.D. Revised and enlarged by Hugh Houston, M.R.C.S.L.—London, 1838. 12mo. pp. 307. 5s.

3. Intermarriage; or the Mode in which, and the Causes why, Beauty, Health, and Intellect result from certain Unions, and Deformity, Disease, and Insanity, from others, &c. Illustrated by Drawings of Parents and Progeny. By Alexander Walker.—London, 1838. 8vo. pp. 442. 14s.

4. Treatise on Physical Education; specially adapted to young Ladies. By A. M. Bureaud Riofrey, M.D. Second Edition, considerably enlarged; with Plates.—London, 1838. 8vo. pp. 574.

5. A Treatise on Inflammation. By James Macartney, M.D. F.R.S. F.L.S. M.R.I.A. F.S.S.L., Professor of Anatomy to the Royal Hibernian Society.—4to. pp. 214; 2 plates. 15s.

6. Medico-Chirurgical Transactions, published by the Royal Medical and Chirurgical Society of London. Second Series. Vol. III.—London, 1838. 8vo. pp. 453. 15s.

7. First Principles of Medicine. By Archibald Billing, M.D. A.M., &c. Third

Edition, considerably enlarged and improved. — London, 1838. 8vo. pp. 288. 10s. 6d.

8. Counter-Irritation, its Principles and Practice, illustrated by one hundred Cases of the most painful and important Diseases effectually cured by external Applications. By A. B. Granville, M.D. F.R.S.—London, 1838. 8vo. pp. 360. 10s. 6d.

9. A Fact in the Natural History of Children, hitherto unobserved; which explains much concerning Infantile Diseases and Mortality. By John Gardner, Surgeon. —London, 1838. 8vo. pp. 23. 1s.

10. General View of the Sources of Difficulty and Fallacy in Diagnosis, (being the Article PRACTICE OF PHYSIC, from the 7th Edition of the Encyclopædia Britannica. By W. Thomson, M.D.—4to. pp. 22.

11. Observations on the Management of Mad-houses; illustrated by Occurrences in the West-Riding and Middlesex Asylums. By Caleb Crowther, M.D., formerly senior Physician to the West-Riding Pauper Lunatic Asylum.—London, 1838. 12mo. pp. 145. 2s. 6d.

12. A Treatise on the Nature and Treatment of Hooping-cough and its Complications, illustrated by Cases: with an Appendix, containing Hints on the Management of Children. By G. H. Roe, M.D. Oxon. Physician to the Westminster Hospital.—London, 1838. 8vo. pp. 258. 8s.

13. The Power, Wisdom, and Goodness of God, as displayed in the Animal Creation; showing the remarkable Agreement between this Department of Nature and Revelation. In a Series of Letters. By C. M. Burnett, Esq., Member of the Royal College of Surgeons.—London, 1838. 8vo. pp. 549; with Plates. 15s.

14. The Cyclopædia of Anatomy and Physiology. Part XIV. GLAND—HEARING (Organ of).—August, 1838. 5s.

15. Transactions of the Medical and Physical Society of Bombay. Vol. I.—Bombay, 1838.

16. Elements of Physiology; being an Account of the Laws and Principles of the Animal Economy, especially in reference to the Constitution of Man. By T. J. Aitkin, M.D. &c. — London, 1838. 8vo. pp. 514. 9s.

17. Report of the Directors of the Dundee Lunatic Asylum, for the year ending May 31, 1838.—Dundee, 1838. 8vo. pp. 36.

18. Statistical Report on the Sickness, Mortality, and Invaliding among the Troops in the West Indies. Prepared from the Records of the Army Medical Department and War-office Returns. By Captain Tulloch.—London, 1838. Folio, pp. 143.

19. On Urinary Diseases, their Consequences and Treatment. By Robt. Willis, M.D. &c.—8vo. pp. xxxvi. and 408.

20. Physician's Report to the Managers of the Lunatic Asylum of Aberdeen, for the year ending 1st May, 1838.—Aberdeen, 1838. 8vo. pp. 14.

21. The Second and Third Fasciculi of Anatomical Drawings, selected from the Collection of Morbid Anatomy in the Army Medical Museum at Chatham.—London, 1834—1838. Atlas; 19 Plates.

22. The Structure, Diseases, and Treatment of the Teeth, considered with a View to the Abolition, in all common Cases, of the pernicious Practice of Tooth-drawing. By Wm. Wardroper, M.R.C.S. — London, 1838. 8vo. pp. 59. 3s.

23. Lectures on Lithotomy, delivered at the New York Hospital, December, 1837. By A. H. STEVENS, M.D., Surgeon of the New York Hospital. —New York, 1838. 8vo. pp. 93; 3 Plates.

24. A Clinical Lecture on the Primary Treatment of Injuries, delivered at the New York Hospital, November, 1837. By A. H. Stevens, M.D. &c.—New York, 1837. 8vo. pp. 34.

25. Remarks on the Preservation of the Teeth from Infancy to Age. By D. W. Jobson, Surgeon-Dentist to the Queen.—London, 1838. 12mo. pp. 94. 1s.

26. The Science of the Cerebro-Spinal Phenomena attempted. By J. S. Waugh, M.D., Annan.—London, 1838. 12mo. pp. 172. 6s.

27. Chemistry of Organic Bodies.—Vegetables. By Thomas Thomson, M.D. F.R.S., Professor of Chemistry in the University of Glasgow, &c.—London, 1838. 8vo. pp. 1076. 24s.

28. An Experimental Essay on the Physiology of the Blood, for which a Gold Medal was awarded by the Medical Faculty of the University of Edinburgh. By Charles Maitland, M.D.—Edinburgh, 1838. 8vo. pp. 90. 2s.

29. The Medical Portrait Gallery; with Biographical Memoirs of the most celebrated Physicians, Surgeons, &c. By T. J. Pettigrew, F.R.S. &c. Parts IV. V. VI. VII., containing Portraits and Memoirs of Blundell, Caius, Morgagni, Radcliffe, Bichât, Cooper, Copland, Cooke, Mead, William Hunter, Jenner, Baron.—Price 3s. each.

30. Analytical Tables, adapted to the Purposes of General and Forensic Chemistry, &c. Translated from the French of Professor Devergie. By M. H. Lynch, M.D. &c. Two large Tables.—Newcastle-on-Tyne, 1838.

31. A Treatise on Neuralgia. By

Richard Rowland, M.D., Physician to the City Dispensary.—London, 1838. 8vo. pp. 173. 6s.

32. Observations on the Preservation of Sight, and on the Choice, Use, and Abuse of Spectacles, Reading-glasses, &c. Third Edition. By J. H. Curtis, Esq., Oculist and Aurist.—London, 1838. 12mo. pp. 74. 1s.

33. Anatomical Tables; containing concise Descriptions of the Muscles, Ligaments, Fasciæ, Blood-vessels, and Nerves. Intended for the use of Students. By T. Nunnely, Lecturer on Anatomy, &c. Leeds. —London, 1838. 12mo. pp. 240. 4s. 6d.

34. An Essay on the Relation between the Respiratory and Circulating Functions. By Charles Hooper, M.D.—Boston, 1838. 8vo. pp. 74.

35. The Boston Medical and Surgical Journal, for July and August, 1838.*

FOREIGN.

1. Ischl e Venezia Memoria del Dott. C. V. L. Brera, &c.—Venezia, 1838. 8vo. pp. 295.

2. Indagini a stabilire quale possa essere il miglior metodo di Cura pel Cholera-Morbus d'oggi giorno, dirette a' giovini medici Italiano. Memoria del Dot. Emiliani, Professore di Medicina nella R. Universita di Modena.—Bologna, 1837. 4to. pp. 100.

3. Ueber Schleim-und Eiterbildung und ihr Verhältniss zur Oberhaut. Von Dr. Henle.—Berlin, 1838. 12mo. pp. 62.

4. Statistica di Coloro che furono presi dal Cholera Asiatico in Roma nell' Anno 1837, umiliata alla Santità di nostro Signore Papa Gregorio XVI. Dalla Commissione Straordinario di Pubblica Incolumità.—Roma, 1838. Fol. pp. 145.

5. Historisch-kritische Darstellung der Pockenseuchen des gesammten Imf-und Revaccinationswesens im Königreiche Würtemberg innerhalb der fünf Jahre, Juli, 1831, bis Juni, 1836. Von Professor Dr. Franz Heim.—Stuttgart, 1838. 8vo. pp. 651.

6. Resultat der Revaccination in dem Königl. Wurtembergischen Militär in den Jahren, 1833, 1834, und 1835. Von Professor Dr. Franz Heim. — Ludwigsburg, 1836. 8vo. pp. 100.

7. Dell' Influenza del Clima nel prevenire e curare le Malattie chroniche, &c. Di Giacomo Clark, M.D. &c. Versione dall Inglese con aggiunte del Dott. Giuseppe Girolami.—Firenze, 1837. 8vo. pp. 260.

8. Saggi Clinici riguardanti forme le piu frequente dell' umano infermare Opera empirico-induttiva del Dott. F. G. Geronimi. Vol. I.—Cremona, 1837. 8vo. pp. 160.

9. Du Varicocele, et, en particulier, de la Cure radicale de cette Affection. Par H. Landouzy.—Paris, 1838. 8vo. pp. 126.

10. De Cholera Asiatica et præsertim de Epidemia Gothoburgensi Adnotationes nonnullæ, quas conscripsit Dr. J. G. Jacobsen.—Upsaliæ, 1838. 8vo. pp. 46.

11. Haandbog i Toxikologien. Af C. Otto, M.D., Professor ved Kjobenhavns Universitet. — Kjobenhavn, 1838. 8vo. pp. 504.

12. Recherches anatomiques sur l'Emphysème pulmonaire. Par le Dr. Lombard, Medecin de l'Hôpital de Genève.—Genève, 1837. 4to. pp. 20.

13. Die Lehre von der Erkenntniss und Behandlung der Lungen- und Herzkrankheiten. Von Dr. P. J. Philipp.—Berlin, 1838. 8vo. pp. 522.

14. Die Behandlung der Hundswuth in politzeilicher, prophylaktischer und therapeutischer Hinsicht. Von Dr. J. R. Sauter.—Konstanz, 1833. 8vo. pp. 179.

15. Over de Overeenkomst en het Verschil Tusschen de Jicht en de Scrophulosis, vooral met Betrekking tot de Longtering; eene Voorlezing door A. A. Sebastian, M.D. Hoogleeraar in de Geneeskunde aan de Hoogeschool te Groningen.—Groningen, 1838.—8vo. pp. 102.

16. Tractatus de Eclampsia. Auctore E. S. Stein, M.D.—Hagæ Comitum, 1837. 8vo. pp. 149.

17. A. A. Sebastian Oratio de Animo et ingenio gentium suis de Medicina meritis evidentissimo, publice habita.—Groningæ, 1837. 4to. pp. 27.

18. Ricerche Patologiche intorno alle Idropi. Del Dottore Giovanni Gandolfi.—Firenze, 1836. 8vo. pp. 295.

19. Memorie della Società Medico-chirurgica di Bologna. Vol. I.—Bologna, 1838. 8vo. pp. 560.

20. Taschenbuch zu gerichtlich-medicinischen Untersuchungen für Aertze, Wundärzte, und Justiz-Beamte. Von J. C. F. Rolffs, M.D., &c.—Köln, 1338. 18mo. pp. 212.

---

* We once more beg to inform our kind friends in America, that books must be sent FREE OF EXPENSE, either to our London publisher, Mr. Churchill, 16, Princes street, Soho, or to our publisher for the United States, Mr. Geo. Adlard, New York. Until an alteration is made in the present rates of postage between Great Britain and foreign countries, conveyance of *Books* by post is quite inadmissible. The two last numbers in our list were sent by post; the one (No. 34) being charged 10*s.* 8*d.*, the other (35) 1*l.* 12*s.*; the sale price of both not being probably more than eighteen-pence!

### INDEX TO VOL. VI.

OF THE

### BRITISH AND FOREIGN MEDICAL REVIEW.

PAGE

ABORTION, singular case of . 236
treatises on . 81
Abscess, penetrating the chest . 234
treatment of . . 156
Absorption, cutaneous, Dr. Madden on 332
Acetate of lead in hernia . . 537
Addison, Dr. on electricity . 72
Adhesion, Sir C. Bell on . . 156
Air into the veins, introduction of 455
experiments on . 517
in respiration, quantity used 580
Albuminous fluids, Dr. Babington on 74
Alcock, Mr. on the Spanish Legion 448
Amussat, M. on air in the veins, 455-527
Amputation, Sir C. Bell on . 166
results of . 561
of the foot . . 535
Amsterdam college and medical school 277
Anatomical Sketches, by Wormald, &c. 202
Anchylosis, new treatment of . 254
Aneurism, popliteal, case of . 67
Arteries, M. Bizot's Researches on 41
Ashwell, Dr. on uterine diseases . 71
Auscultation, artificial . 532
Babington, Dr. on albuminous fluids 74
Barlow, Dr. his address . . 570
Dr. H. on the causes of disease 201
Barton, Dr. his treatment of anchylosis 254
Baron, Dr. his Life of Dr. Jenner 477
Baths of hemlock in skin diseases 538
Beaumont, Dr. on digestion . 172
Belladonna in typhus fever . 554
Bell, Sir C. his institutes of Surgery 154
Belmas, Dr. on the cure of hernia . 341
Berres, Dr. on the nerves . 393
Bidder, Dr. his neurological researches 204
Bile, healthy and calculous, analysis of 247
Bizot, M. his researches on the heart 41
Bladder, diseases of, Mr. Coulson on 509
Blandin, M. on amputation of the foot 535
Blood-globules, on the motions of the 219
Blood, analysis of . . 248, 443
chemistry of . . 431
Bloodletting, on the abuse of . 515
Blizard, Mr. obituary of . 288
Bonnet, M. on the cure of hernia 345
Books received for review . 291, 594

PAGE

Botany, British, Dr. Macreight on 511
Bougies, new material for . 259
Bouillaud, M. on rheumatism . 352
on air in the veins 517
Brain, case of tumour of . 226
on the structure of . 393, 400
exploration of . . 144
Bright, Dr. on abdominal tumours 72
British Legion in Spain, history of 448
Association, report of the 573
Bronchitis, use of chlorine in . 533
Bronchocele, relative prevalence of 27
Burdach, M. on the nerves . 394
Burke, Mr. on fractures . 308
Burne, Dr. on hydrophobia . 268
Cancer, relative prevalence of . 20
of the uterus, Dr. Churchill on 90
Capillary vessels, anatomy of . 218
Carbonic acid, murder by . 241
Carbonate of iron in hooping cough 222
Carlsbad waters in tic douloureux 511
Carmichael, Mr. on venereal ulcers 564
Carotid artery, ligature of . 233
Carpenter, Mr. on vital phenomena 553
on Physiology 553, 590
Cataract, new mode of diagnosticating 231
M. Maunoir on . 50
Cervix uteri, inflammation of . 89
Chase, Dr. on the radical cure of hernia 341
Cheltenham, medical topography of 384
Chemistry, Mitscherlich's treatise on 505
Children, Dr. Ure on diseases of . 516
Chlorine in bronchitis . . 533
Cholera, statistics of, in Berlin . 240
on the laws of . . 576
Chomel, M. on gout and rheumatism 352
Churchill, Dr. on diseases of females 86
Clavicle, luxation of, downwards . 229
Climate of Exeter . . 383
influence of, on health . 148
Clot-Bey on Medicine in Cairo . 592
Club-foot, Sir C. Bell on . 163
cured by operation . 272
Cock, Mr. on the pneumo-gastric nerve, 60
Colchicum in rheumatic gout . 556
Colquhoun, Mr. on a case of idiocy 122
Combe, Dr. on digestion . . 172

PAGE
Conquest, Dr. on tapping the head 265
Cooper, Mr. B. his surgical cases . 66
Copland, Dr. on abortion . 81
Copaiva, new mode of administering 557
Cormack, Dr. on air in the veins . 455
Coulson, Mr. on diseases of the bladder, 509
Counter-irritation, Dr. Granville on 498
Creosote, on the topical use of 270, 235
Cross, Dr. on delirium tremens . 320
Croup, cauterization in . 530
Crystalline lens, anatomy of . 205
Cyanosis from absence of one lung 220
Cystitis, use of nitrate of silver in 565
D'Amador, M. on pathological anatomy, 293
Davy, Dr. on the vesiculæ seminales 551
Death after wounds, not from . 540
Deckmann, Dr. obituary of . 290
Delirium tremens, works on . 320
statistics of . 323
description of 325
treatment of . 328
Dendy, Mr. on cutaneous diseases 425
Denis, M. on the chemistry of the blood, 431
Desgenettes, M. obituary of . 290
Deventer, college of . . 280
Dewees, Dr. on abortion . . 81
Diagnosis, M. Piorry on . 128
Dick, Dr. on porrigo . . 425
Dieffenbach on ruptured perinæum 536
Digestion, artificial, galvanism in . 527
experiments on . 172
Disease, moral causes of . . 201
Diseases of the Landsend and Worcester 8
Dislocation of the humerus . 231
of the tarsus . 538
Donné, Dr. on the milk of nurses . 181
Dropsy, relative prevalence of . 17
on elder berries in . 221
Dropsical effusions, urea in . 249
Dugès, M. on abortion . . 81
Dunglison, Dr. on polypus of the heart 547
Ear, Mr. Webster on the . 199
Egypt, influence of its climate on health, 151
Ehrenberg, M. on the brain . 393
Electricity, animal, remarkable case of 249
as a remedy . . 72
Elm-bark, bougies &c. made of . 259
Estlin on vaccine virus . 591
Embryotomy, remarkable case of . 236
Emmert, M. on the nerves . 394
Emphysema of the lungs . 28
Empyema, case of . . 266
Encephalitis, symptoms of . 144
Epidemic constitution, on animals 220
Epiglottis, acute inflammation of 261
Epilepsy, epidemic in schools . 531
Eruptions, relative prevalence of . 27
Evans, Mr. on copaiva . 559
Exeter, medical topography of . 382
Experiments on air in the veins . 517
Eye, on disease of . . 73
turpentine in diseases of . 533
Farcy in man . . . 114

PAGE
Farr, Mr. on the laws of cholera . 576
Feminiscence . . . 75-79
Fenner, Mr. on abdominal pressure 567
Fergus, Dr. obituary of . 288
Fevers, relative prevalence of . 9
Fever, typhus, Dr. Graves on . 554
statistics of . . 556
puerperal, Dr. Gruber on . 539
Finck, Dr. on the cure of hernia 341
Foot, amputation of . . 535
dislocation of . . 538
Fractures, statistics of, in America 258
in London 310
on the treatment of . 308
Fractures of the ribs, spontaneous 227
Franecker, college of . . 280
Frostbite, amputation from . 550
Galen, analysis of his writings . 385
Galvanism, effect of, in digestion 527
Ganglions, structure of . . 404
Gangrene, senile . . 157
of the hand . 66
Gastric juice, experiments on . 172
nature of . 177
Gerdy, M. on the cure of hernia . 348
Glands, sudorific, account of . 514
Glanders in man . . 113
Glaucoma, Dr. Mackenzie on . 274
Glottis, scalded . . 270
Glover, Mr. on the rete mucosum 574
Gout, on the nature and cure of . 352
Granville, Dr. on counter-irritation 498
Graves, Dr. on the pupil in fever . 554
Greenhow, Mr. on neuralgia . 573
Groningen, University of . 277
Gulliver, Mr. on suppuration . 553
Gurlt, Dr. his physiology . 513
Guy's Hospital Reports . 60
Hallmann, M. on the temporal bone 28
Hammick, Dr. his Mitscherlich . 505
Hancock, Mr. his translation of Velpeau 193
Handyside, Dr. his case of suicide 473
Harrison, Dr. obituary of . 289
Health, Sir A. Carlisle on . 186
Heart, exploration of . . 132
researches on . . 41
rheumatic affections of . 362
hypertrophy of, treatment of 387
diseases of, prevalence of . 20
polypus of . . 547
Hernia, on acetate of lead in . 537
Hemorrhage, Sir C. Bell on . 157
Hemlock baths in diseases of the skin 538
Hernia, improved mode of reducing 558
on the radical cure of . 341
Mr. Hilles on . . 203
cases of . . . 68-70
Hilton, Mr. on the laryngeal nerves 61
Hindoo medicine, Dr. Royle on 506
Höegh, Dr. on delirium tremens . 320
Holland, present state of medicine in 275
Holst, Dr. on tumour of the brain 226
Hooping-cough, carbonate of iron in 222

PAGE
Hunger, theory of . . 179
Hunterian oration, Mr. Travers's 191
Hunter, John, his anticipations 192
Hutchinson, Mr. on tic douloureux 511
Hydrocephalus, on tapping the head in 265
Hydrophobia, cases of . . 268
Hysteria, Mr. Prichard on . 512
Idiocy, cases of alleged . 122
Immoveable apparatus in fractures 310
objections to 315
India, mortality of troops in . 151
Inflammation, Mr. Macilwain on . 122
Inflammation of the bladder, Mr. Coulson on . . . 510
Intestinal diseases, prevalence of . 26
Intestinal glands, anatomy of . 218
Intussusception, treated by inflation 274
Inversion of the uterus . 96, 236
Ipecacuanha, new preparation of . 247
Jenner, Dr. his life, by Dr. Baron 477
Journals, American and Colonial . 260
Key, Mr. his case of hernia . 70
Kidd, Professor, on the works of Galen 385
Krause, Dr. his treatise on anatomy 204
anatomical observations 218
Lallemand, M. on cystitis . 565
Landsend, medical topography of 3
Langley, Mr. on puncturing small-pox 563
Larrey, M. his treatment of fractures 312
Laryngotomy, case of . . 270
Laryngeal nerves, Mr. Hilton on . 61
Le Canu, M. on the blood . 431
Lee, Dr. on abortion . . 81
Leeches, Prussian regulations regarding . . . . 594
Legs, amputation of both . 550
Leyden, university of . . 279
Life, Mr. Macilwain on . 100
Liquor amnii, analysis of . 248
Liver, exploration of . . 140
London, medical topography of . 187
Lonsdale, Mr. on fractures . 308
Lotion, Dr. Granville's antidynous 500
Louis, M. on emphysema of the lungs 28
Lungs, diseases of, relative prevalence of 20
exploration of . . 137
of infants, experiments on 62
Lunatic Asylums, statistics of 239-240
Lusus naturæ, remarkable . 381
Lymph, analysis of . . 544
Macilwain, Mr. on medicine, &c. 98
Mackenzie, Dr. on glaucoma . 274
Macreight, Dr. his British botany 511
Madden, Dr. on cutaneous absorption 332
Malgaigne, M. on dislocations . 231
Mammalia, domestic, physiology of 513
March, Dr. on the epiglottis . 261
Massalien, Dr. on the facial nerves 200
Maunoir, M. on cataract . 50
Medical institutions in Cairo . 592
Medicine and surgery one science 98
Medical portrait gallery . . 194
relief under the new poor-law 586

PAGE
Medicine, on the improvement of 196
is it a science? . 102
Hindoo, account of . 506
Mehliss, Dr. on virilescence, &c. 75
Memoirs of the Paris Society . 28
Mouth, exploration of . 138
Menstruation . . . 92
Meyer, Dr. on epidemic epilepsy . 531
Milk of nurses, examination of . 181
Mitscherlich, Dr. his chemistry . 505
Moles . . . . 92
Morphium, its use in hydrophobia 268
Mortality, relative rate of . 6
Morgan, Mr. on ophthalmia . 74
Mucus, an analysis of . 546
Negroes, mortality of . . 150
Nerve, facial, account of . 300
Nerves, termination of . . 414
wounds of . 170, 271
on the structure of . 393
functions of the eighth pair of 574
Neuralgia, on mercury in . 573
relative prevalence of . 14
Niuwenhuijs, Dr. on Dutch medicine 275
Nitrate of silver in chronic cystitis 565
Nitrogen, its influence on plants . 552
Nurses, quality of their milk explained 181
Obituary of Dr. Sims . 594
O'Bryen, Dr. on chronic cystitis 565
O'Beirne, Dr. on strangulated hernia 558
Oliver, Dr. his physiology . 195
Ophthalmia in the Belgian army . 197
Orchitis, M. Marc d'Espine on . 59
Œsophagus, stricture of . 165
Os uteri, cartilaginous, incision of 235
Otaphone, an instrument for the deaf 199
Owen, Prof. on the teeth . 578
Paraplegia . . 261
Pathological anatomy, influence of 293
Petrequin, M. on artificial auscultation 532
Perineum, rupture of . . 536
Perforation of the stomach . 221
Pettigrew, Mr. his portrait gallery 194
Phillips, Mr. on amputation . 561
Phlebitis, fatal case of . . 228
Phthisis pulmonalis, early signs of 534
epidemic . 227
Physiology, Dr. Oliver's first lines of 195
Pineal gland, dropsy of . 268
Piorry, M. on diagnosis . 128
Plants, effect of nitrogen on . 552
Pneumonia, case of . . 547
Pneumogastric nerve, Mr. Cock on 60
Poisoning by oxide of zinc . 221
Polypus, bronchial . . 268
uteri . . 93
Portugal, state of medicine in . 284
Porrigo, Dr. Dick on . . 425
Pregnancy, extra-uterine . 252
Pressure in venereal phagedæna 564
Pressure after delivery . . 567
Prichard, Mr. on hysteria . 512
Prolapsus uteri . . 96

PAGE
Prolapsus vaginæ . . 93
Provincial Association, meetings of 287, 569
transactions of 1, 380
Pupil in typhus, Dr. Graves on the 654
Purkinje on artificial digestion . 527
Pus, evacuations of, in hepatitis . 351
chemistry of . . 646
Ranula . . . . 164
Rasori, obituary of . . 289
Rayer, M. on glanders in man . 113
Reflex motions, Dr. Volkmann on 211
Reid, Dr. on the respiration . 580
Dr. J. on the 8th pair of nerves 574
Rejuvenescence, Dr. Mehliss on 75, 80
Remak on the structure of the nerves 394
Reports of infirmaries, plan of . 381
Respiration, quantity of air used in 580
Rheumatism, on the nature and cure of 352
chronic . 371
muscular . . 354
visceral . 373
articular . . 358
treatment of, by bloodletting 369
relative prevalence of 14
Rheumatic gout, treatment of . 556
Ribs, excision of . . 548
Rigg, Mr. on the growth of plants 552
Royle, Dr. on Hindoo medicine . 506
Rupture of the uterus . 539
Ryan, Dr. on abortion . . 81
Saucerotte, M. on pathological anatomy, 293
Sanson, M. on cataract . 231
Salter, Mr. on hypertrophy . 387
Scrofula, relative prevalence of . 19
Sir A. Carlisle on . 188
Semen, human, Dr. Davy on . 551
Semeiology, M. Piorry on . 128
Seutin, M. his treatment of fractures 314
Serous membranes, adhesion of . 232
Severn, Vale of, topography of . 4
Sewell, Dr. on amputation in frostbite 550
Shapter, Dr. on Exeter . . 382
Siebold, Dr. on abortion . 81
Skin, diseases of, in children . 425
anatomy of . . 333
hemlock baths in diseases of 538
Small-pox same origin as cow-pox 482
on puncturing . 563
Speculum, use of . . 91
Spermatorrhœa, treatment of . 532
Spine, diseases and injuries of . 161
Spleen, exploration of . 160
Statistics, medical . . 1
Stammering, on the cure of . 253
St. Martin, his curious case . 173
Stomach, exploration of . . 139
Stone in the bladder, case of . 70
Strangulation, new mode of treating 558
Strychnine, M. Bally on . 225
Suckow, Dr. on semeiotics . 147

PAGE
Suicide, statistics of . . 241
Sulphuric acid, absorption of . 244
Suppuration, Mr. Gulliver on . 553
Surgery, Sir C. Bell's institutes of 154
Surgery, is it a science? . 102
Synovial effusions, treatment of . 224
Syphilis, endemic in Lithuania . 223
Sir C. Bell on . 171
Tapping the head in hydrocephalus 265
Tartar emetic in synovial swellings 224
Taylor, Mr. on the lungs of infants 62
Teeth, Prof. Owen on the structure of 578
Temperature of the body . 142
Temporal bone, anatomy of . 201
Thomson, Dr. S. on climate . 148
Dr. Th. his oration . 196
Dr. A. on fever . 556
Thymus gland, anatomy of . 218
Tic douloureux, Sir C. Bell on . 169
Mr. Hutchinson on 511
Tin hydrochlorate, medicinal use of 530
Topography, medical . . 1
Transactions of the Provincial Association . . . 1, 380
Travers, Mr. his Hunterian oration 191
Treviranus on the structure of the retina, 394
Tumours, abdominal, Dr. Bright on 72
Ulcers, excision of . . 230
description of . 159
venereal, on pressure in . 564
Ure, Dr. A. on the diseases of children 516
Urethra, structure of . . 165
Uterus, rupture of . . 539
Dr. Ashwell on diseases of 71
Uterine diseases, relative prevalence of 26
Utrecht, university of . . 277
Vaccination, history of . 478
Vaccine fluid, examination of 247, 546
Vaccine virus, M. Estlin on . 591
Valentin on the termination of nerves 394
Varicocele, new mode of treating . 274
Veins, varicose, treatment of . 257
introduction of air into 455, 517
substances introduced into 577
Velpeau on air in the veins . 455
his treatment of fractures 315
his anatomy of regions . 193
Virilescence, Dr. Mehliss on . 75
Vital phenomena, Mr. Carpenter on 553
Volkmann, Dr. on reflex motions . 211
Vulva, affections of . . 87
Ward, Dr. on empyema . . 266
Warren, Dr. on excision of the ribs 548
Werneck, Dr. on anatomy of the eye 205
Wetzlar, Dr. on bloodletting . 518
Wigan, Mr. on rheumatic gout . 556
Wormald, Mr. his anatomical sketches 202
on varicocele . 274
Wounds, death after, but not from 540
Wound of the tongue, fatal . 69

END OF VOL. VI.

PRINTED BY C. ADLARD, BARTHOLOMEW CLOSE.

